# Adaptive Mechanisms Make Lupin a Choice Crop for Acidic Soils Affected by Aluminum Toxicity

**DOI:** 10.3389/fpls.2021.810692

**Published:** 2022-01-05

**Authors:** Miguel A. Quiñones, M. Mercedes Lucas, José J. Pueyo

**Affiliations:** Institute of Agricultural Sciences (ICA), Consejo Superior de Investigaciones Científicas (CSIC), Madrid, Spain

**Keywords:** lupin, *Lupinus albus*, acidic soils, aluminum, cluster roots, *Bradyrhizobium*

## Abstract

Almost half of the world’s agricultural soils are acidic, and most of them present significant levels of aluminum (Al) contamination, with Al^3+^ as the prevailing phytotoxic species. Lupin is a protein crop that is considered as an optimal alternative to soybean cultivation in cold climates. Lupins establish symbiosis with certain soil bacteria, collectively known as rhizobia, which are capable of fixing atmospheric nitrogen. Moreover, some lupin species, especially white lupin, form cluster roots, bottlebrush-like structures specialized in the mobilization and uptake of nutrients in poor soils. Cluster roots are also induced by Al toxicity. They exude phenolic compounds and organic acids that chelate Al to form non-phytotoxic complexes in the rhizosphere and inside the root cells, where Al complexes are accumulated in the vacuole. Lupins flourish in highly acidic soils where most crops, including other legumes, are unable to grow. Some lupin response mechanisms to Al toxicity are common to other plants, but lupin presents specific tolerance mechanisms, partly as a result of the formation of cluster roots. Al-induced lupin organic acid secretion differs from P-induced secretion, and organic acid transporters functions differ from those in other legumes. Additionally, symbiotic rhizobia can contribute to Al detoxification. After revising the existing knowledge on lupin distinct Al tolerance mechanisms, we conclude that further research is required to elucidate the specific organic acid secretion and Al accumulation mechanisms in this unique legume, but definitely, white lupin arises as a choice crop for cultivation in Al-rich acidic soils in temperate climate regions.

## Introduction

White lupin (*Lupinus albus* L.) is a singular legume with increasing value both as an agronomic and a horticultural crop (Jansen, 2006). Lupins seeds present protein contents close to 40% and are among the most protein-rich plant products, making this legume an excellent protein crop and a viable alternative to soybean cultivation in cold climates (Lucas et al., 2015; De Ron et al., 2017). As a horticultural crop, white lupins play a role in ecosystem services as honey plants and annual ornamentals (Jansen, 2006). White lupin has a notable tolerance to abiotic stresses (Fernández-Pascual et al., 2007), and a considerable potential as a tool for the recovery of degraded soils and phytoremediation of toxic metal-polluted soils (Coba de la Peña and Pueyo, 2012; Quiñones et al., 2013, 2021). Lupins establish symbiosis with soil bacteria leading to the formation of nitrogen-fixing root nodules. Moreover, several lupin species are capable of developing the so-called cluster roots (CR) (Lambers et al., 2003; Pueyo et al., 2021). P or Fe deficiency and Al toxicity, which often coexists with P deficiency in globally-distributed acidic soils, induce the formation of CR, which promote mobilization of P and other nutrients by secretion of organic acids (OAs), protons, phosphatases and phenolic compounds (Sun et al., 2016). These distinctive traits confer white lupin the competence to grow in N- and P-deprived soils, mobilizing other nutrients, which are unavailable to most plants. Unlike other legumes, lupins can grow optimally at pH > 4.0, and sub-optimally at even lower pHs (Nelson et al., 2001; Zavalin et al., 2019).

**FIGURE 1:**
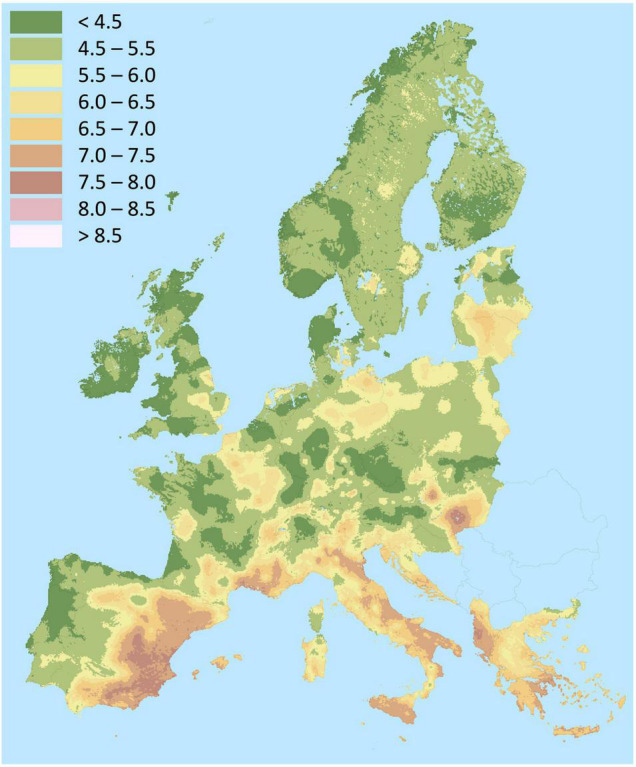
Map of soil pH in Europe. Source: Land Resources Management Unit, Institute for Environment and Sustainability, European Soil Data Centre (ESDAC), esdac.jrc.ec.europa.eu, European Commission, Joint Research Centre (Panagos et al., 2012).

It is estimated that roughly 30% of the world’s soils are acidic (Zheng, 2010). In many areas of the Pacific Ring of Fire, volcanic-originated soils are particularly acidic, Al-toxic and P-deficient Andosols that comprise approximately 50% of arable land in Chile (Redel et al., 2016). Acidic soils are also predominant in Europe, mostly in the Northern regions (Figure 1). Soil acidification is accentuated by some agricultural practices (Moore et al., 2001). Climate change and the fluctuations in rainfall patterns also contribute to soil acidification. While other metals are more toxic, aluminum constitutes the main problem of acidic soils, which depends on soil pH, but also on factors such as organic matter, phosphates, fluorides, sulfates or ionic strength (Carr and Ritchie, 1993). Acidic soils are usually rich in clay minerals such as iron oxides, kaolinite, montmorillonite and illite that trap P and render it inaccessible for plants (Zheng, 2010; Eriksson et al., 2016). Therefore, P deficiency and Al toxicity coexist in acidic soils severely hampering crop production. Lime and P applications are effective in the short term, they elevate the soil pH, reduce Al solubility, which is also displaced from exchangeable sites by Ca, and increase P availability. However, the soil buffering capacity can diminish the effects of the amendments. Acidification is increased by factors such as acid rain and application of ammonium-based fertilizers, and amendments need to be reapplied, which might not be sustainable in the long term (Zheng, 2010). A crop such as lupin, which tolerates Al toxicity and displays mechanisms to fix atmospheric nitrogen and mobilize P and other nutrients in nutrient-deprived acidic soils, arises as an ideal option for sustainable agriculture in temperate zones worldwide.

In plants, Al can cause cytotoxic inhibition, damages to cell structures and eventual suppression of plant growth and crop yield (Andersson, 1988). Al toxicity symptoms include disturbance of the plasma membrane, decrease in water uptake, damage to photosynthesis, and generation of reactive oxygen species (ROS) (Wei et al., 2021). Al inhibits auxin transport, induces ethylene production and reduces root growth (Chauhan et al., 2021; Wei et al., 2021). Al causes epidermal cell death, decreasing cell extensibility and conductivity (Chauhan et al., 2021). Al interferes with the uptake, transport and metabolism of multiple mineral nutrients (Andersson, 1988). It alters the expression of numerous genes, it affects the ability of DNA to replicate, and disturbs signal transduction pathways (Sade et al., 2016). Some plants have developed Al tolerance through root secretion of chelating compounds. Several transporters involved in Al resistance mechanisms belong to the multidrug and toxic compound extrusion (MATE), the aluminum-activated malate transporter (ALMT), the natural resistance associated macrophage protein (NRAMP), the ATP-binding cassette (ABC), and the aquaporin families (Wei et al., 2021). OAs and phenolic compounds bind Al and exclude it from the cell and/or sequester the resulting complexes into the vacuole or the cell wall (Sade et al., 2016). A fine regulation of the response to Al stress is achieved by the involvement of phytohormones, Ca^2+^ signaling pathways, transcription factors and miRNAs (Matsumoto, 2000).

## Physiological Alterations and Specific Mechanisms Involved in Lupin Tolerance to Aluminum

White lupin response to Al toxicity involves physiological and morphological modifications and mechanisms that differ from other plants, including other legumes. Due to its high tolerance, Al toxicity has been scarcely investigated in lupin, but at high enough concentrations, Al can cause a suppression of vegetative growth, with decreases in shoot and root biomasses (Hemada et al., 2020). Lupin CR exudates limit the entrance of Al into the roots through the formation of non-toxic complexes (Dakora and Phillips, 2002). Malate, citrate and oxalate are involved in Al resistance in plants (Ma, 2000; Ma et al., 2001). Malate and citrate are secreted from root tips and regulated by specific transporters (Sharma et al., 2016; Wang et al., 2017); however, oxalate secretion transport pathways are mostly unknown (Lv et al., 2021). CR exudates consist mainly of OA anions (Dessureault-Rompré et al., 2007), protons, phenolic compounds and phosphatases (Gerke et al., 1994; Gilbert et al., 1999; Lamont, 2003) that generate changes in the rhizosphere and mobilize nutrients and toxic metals from insoluble phosphates (Dakora and Phillips, 2002). OAs act as detoxifying agents by chelating Al into non-phytotoxic forms (Dakora and Phillips, 2002). OA anion exudation by lupin CR has a strong effect on cation concentration in the rhizosphere, but this does not occur through changes in pH, but through metal complexation (Dessureault-Rompré et al., 2008), ligand exchange (Violante et al., 1991; Geelhoed et al., 1998) and dissolving organic matter (Kaiser and Zech, 1997; Beck et al., 1999). P deficiency and Al toxicity frequently coexist on acid soils, suggesting that response mechanisms might have evolved to adapt to both stresses through common regulation pathways (Sun et al., 2016).

Enzymes of the TCA cycle, the glycolysis pathway and the glyoxylate cycle are involved in OA biosynthesis (Igamberdiev and Eprintsev, 2016). The conversion of pyruvate to malate, phosphoenolpyruvate to oxaloacetate and oxaloacetate to malate are Al-induced in soybean (Dong et al., 2004). Al toxicity increases malate exudation in *Lupinus pilosus* (Ligaba et al., 2004). The activities and gene expression levels of citrate synthase, malate dehydrogenase and phosphoenolpyruvate carboxylase have been reported to increase in white lupin CR under P deficiency (Johnson et al., 1994; Uhde-Stone et al., 2003). Aconitase and malic enzyme activities are also affected by Al stress in soybean (Xu et al., 2010; Zhou et al., 2018).

Differences exist between P deficiency- and Al toxicity-induced citrate secretion (Wang et al., 2007). P deficiency induces citrate release from mature CR, while Al triggers exudation from the subapical root zones of lateral roots and from mature and senescent CR. Al-induced citrate exudation is inhibited by P limitation at the seedling stage, but is stimulated at later stages. Citrate secretion is dependent on plasma membrane H^+^-ATPase in lupin under P deficit (Tomasi et al., 2009), and H^+^-ATPase also participates in regulating Al-activated citrate exudation in different legumes (Shen et al., 2005; Guo et al., 2013). However, Al-activated lupin citrate secretion is independent of plasma membrane H^+^-ATPase. Citrate secretion is coupled with K^+^ efflux in P-deficient lupin plants, which may compensate H^+^ efflux and keep the charge balance, but this Al-induced K^+^ efflux is independent of Al-induced citrate exudation (Zeng et al., 2013). OAs release is mediated by the activation of transport systems and the expression of stress-induced specific genes (Ma, 2000). In white lupin, LaALMT1 contributes to malate, but not citrate release (Zhou et al., 2020). Contrary to some ALMT homologs in other species, LaALMT1 expression, which is involved in root-to-shoot translocation, is not stimulated by Al. Other LaALMT homologs, which are upregulated by Al, might have a role in lupin Al-tolerance (Zhou et al., 2020). MATE/AACT/DTX transporters might be involved in Al-stimulated citrate release in lupin (Chen and Liao, 2016; Zhou et al., 2020). Besides malic and citric, oxalic, fumaric, malonic and α-ketoglutaric acids have been identified in the rhizosphere of white lupin (Mimmo et al., 2008), and might contribute to lupin Al tolerance. In certain plants, oxalate rather than citrate or malate is involved in Al tolerance (Yang et al., 2011; Lv et al., 2021), but the regulation of oxalate secretion requires further study. Recently, a potential mechanism for Al tolerance in alfalfa has been described that involves oxalate secretion and ABA-mediated signaling (Lv et al., 2021). Flavonoids exuded by lupin roots play a key role in the establishment of symbiosis by inducing the expression of rhizobial *nod* genes (Cooper, 2007), but flavonoids secreted by CR do not differ from those secreted by regular roots (Weisskopf et al., 2006; Cesco et al., 2010). Secretion of flavonoids by CR precedes that of OAs (Cesco et al., 2010; Chen and Liao, 2016), and they can chelate the metal ions that are solubilized by OAs (Weisskopf et al., 2006; Wei et al., 2021).

**FIGURE 2:**
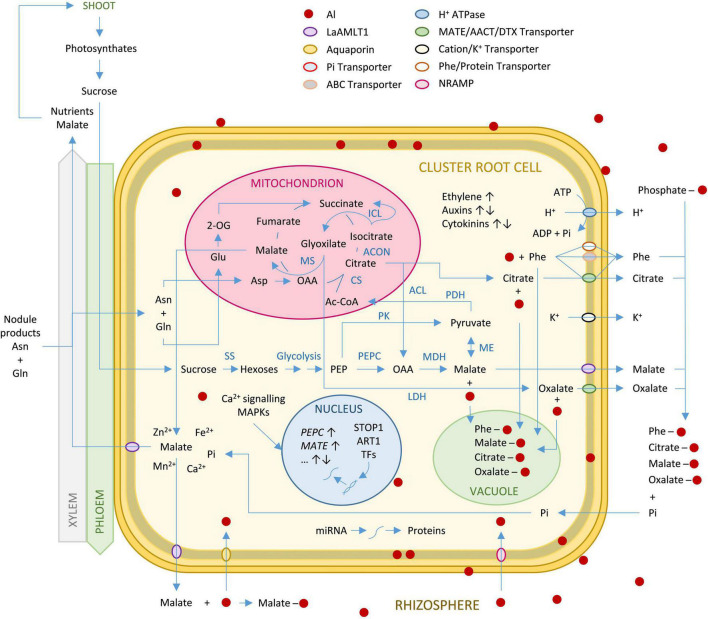
Simplified metabolic pathways in a white lupin cluster root cell in response to Al stress. Aluminum present in the rhizosphere enters the cells primarily through aquaporins and NRAMP transporters. Through the action of Ca^2+^ signaling pathways, transcription factors and miRNAs, Al induces replication of DNA that leads to activation of the TCA cycle, the glycolysis pathway and the glyoxylate cycle. This leads to the accumulation of organic acids and phenolic compounds. Activation of specific transport systems (MATE, ALMT, ABC-transporters) leads to the exudation of these compounds into the rhizosphere. OAs and phenolic compounds chelate Al cations outside and inside the cells. Intracellular complexes are accumulated in the vacuole. Extrusion of H^+^ and K^+^ contribute to the maintenance of the membrane potential. Ethylene production increases, while auxin and cytokinin levels increase or decrease depending on the developmental stage and the different root zones. Abbreviations: 2-OG, 2-oxoglutarate; AACT, aluminum-activated citrate transporter; ABC, ATP-binding cassette; ACON, aconitase; AMLT, aluminum-activated malate transporter; ACL, ATP citrate lyase; ART1, Al Resistance Transcription Factor 1; Ac-CoA, Acetyl CoA; Asn, asparagine; Asp, aspartic acid; CS, citrate synthase; DTX, detoxification transporter; Gln, glutamine; Glu, glutamic acid; ICL, isocitrate lyase; LDH, lactate dehydrogenase; MAPK, mitogen-activated phosphate kinase; MATE, multidrug and toxic compound extrusion; MDH, malate dehydrogenase; ME, malic enzyme; MS, malate synthase; NRAMP, natural resistance associated macrophage protein; OAA, oxaloacetate; PDH, pyruvate dehydrogenase; PEP, phosphoenolpyruvate; PEPC, PEP carboxylase; Phe, phenolic compounds; PK, PEP kinase; SS, sucrose synthase; STOP1, Transcription factor sensitive to protein rhizotoxicity 1; TFs, transcription factors.

Several genes involved in auxin and ethylene signaling have been shown to control both P deprivation and Al stress suggesting a P-Al signaling crosstalk (Sun et al., 2016). Phytohormones have different roles in different plants under Al stress (Rangel et al., 2007; Rademacher et al., 2011; Yang et al., 2014; Sun et al., 2016). Auxin response factors (ARF) in roots are regulated by Al-modulated miRNAs (Wang et al., 2005; Liu et al., 2007). Ethylene biosynthesis genes are induced by Al (Sun et al., 2016), and ACC synthase and ACC-oxidase activities correlate with Al-induced ethylene evolution (Sun et al., 2007; Swarup et al., 2007). Ethylene triggers auxin synthesis in the transition zone of the root apex, inhibiting root growth (Yang et al., 2014). In fact, CR regulatory components are common to regular roots (Secco et al., 2014). CR formation is initiated by the same phytohormones and transcription factors employed by simple-patterned roots (Coudert et al., 2010; Petricka et al., 2012). PIN and LAX auxin carriers (Billou et al., 2005; Grieneisen et al., 2007; Swarup et al., 2008), or Aux/IAA proteins working in combination with ARFs (Vanneste and Friml, 2009) occur in both CR and regular root tips. Root-originated auxin has also been detected in lupin CR (Meng et al., 2013). Transcription factors involved in root meristem function and CR development are also coincident (Galinha et al., 2007; Carlsbecker et al., 2010; Secco et al., 2014). A simplified representation of the molecular mechanisms in lupin CR in response to Al toxicity is presented in Figure 2.

## Role of Rhizobia in Lupin Aluminum Tolerance

Plant growth-promoting rhizobacteria (PGPR) can positively affect Al stress tolerance reducing Al uptake and accumulation, decreasing lipid peroxidation and enhancing root development through ACC deaminase and IAA production or induction of plant genes. Some rhizobia possess PGPR properties, produce exopolysaccharides and secrete OAs, phenolic compounds and sugars that can chelate Al (Kang et al., 2021). Malate-chemotaxis is a mechanism for the recruitment of beneficial rhizobacteria (Rudrappa et al., 2008). Lupin-nodulating rhizobia belong primarily to the *Bradyrhizobium* genus, although several other bacteria have been shown to form efficient symbiosis with lupins (Msaddak et al., 2021). Rhizobial entry occurs at the junction between a root hair and an adjacent epidermal cell (González-Sama et al., 2004), and rhizobia are distributed between the daughter cells (Fedorova et al., 2007; Coba de la Peña et al., 2018), while endoreduplication processes take place (González-Sama et al., 2006). Al can severely inhibit rhizobial growth and decrease nitrogenase activity (Lesueur et al., 1993; Arora et al., 2010). However, highly Al-tolerant strains have been characterized (Vargas et al., 2007; Ferreira et al., 2012; Kang et al., 2021). Strains isolated from acidic Al-contaminated soils show a high production of exopolysaccharides and a decreased outer membrane permeability (Ferreira et al., 2012). Al stress also promotes the production of Al-chelating siderophores (Roy and Chakrabartty, 2000). Overall, *Bradyrhizobium* species are more tolerant to acid and Al stress than fast-growing species (Flis et al., 1993). Several mechanisms have been proposed for acid tolerance, including changes in lipopolysaccharide composition of the outer membrane or polyamine accumulation (Chen et al., 1993; Ferreira et al., 2012; Jaiswal et al., 2018). High exopolysaccharide production by lupin-nodulating bradyrhizobia has been proposed to prevent Hg toxicity (Arregui et al., 2021; Quiñones et al., 2021). Physical sequestration, exclusion and complexation mechanisms contribute to reduce metal availability (Rajkumar et al., 2010). Metals bind to carboxyl and hydroxyl groups on the cell surface, and to microbial secreted substances (Pulsawat et al., 2003). Many lupin species and native soil rhizobia are naturally resistant to low pH and Al toxicity, and tolerant rhizobia favor plant establishment and development (Sprent, 2009). Interestingly, white lupin appears to have developed a strategy to avoid excessive OAs microbial degradation (Weisskopf et al., 2006). The stability of carboxylates in the soil is key to maintain P acquisition and counteract Al toxicity. This is achieved by a temporal regulation of the secretion of isoflavonoids, antifungal cell wall-degrading enzymes, chitinase and glucanase prior to OAs excretion, thus reducing microorganisms population and preventing OA degradation (Neumann et al., 1999; Burzyński et al., 2000; Zhu et al., 2005).

## Discussion

The gradual acidification of the world’s cultivated soils is predominantly driven by agricultural practices and global change. A decrease in pH leads primarily to the complexation of P, rendering it unavailable to plant roots, and to the release of phytotoxic Al cations. Alkaline amendments, such as lime, temporarily increase soil pH. However, in the long term, they might contribute to surface water eutrophication. Sustainable alternatives are needed to confront this global problem. In acidic soils, plants can prevent Al toxicity in a zone around the root tip, by avoiding its entrance into root cells mainly through the exudation of AOs (Wei et al., 2021). Lupin CRs are specialized organs composed of hundreds of small rootlets with active root tips that are able to exudate AOs and other metabolites (Pueyo et al., 2021 and references therein). We propose that CR multiply the defense mechanisms present in other plants, thus significantly increasing Al tolerance. Cultivation of lupin, a plant that is able to cope with Al toxicity and possesses the ability to fix nitrogen in symbiosis with rhizobia and the capacity to mobilize P and other various nutrients through CR exudation, is able to contribute to a sustainable management of agricultural soils affected by low pH and Al toxicity, without compromising crop production. Additionally, the elevated protein content in lupin seeds makes it an extremely valuable crop due to the increasing demand of plant protein. Accurate studies on Al content in lupin organs grown in acidic soils with different soil textures and different Al speciation must be performed to ensure the seed protein safety used for feed and food.

Lupins are cultivated worldwide, but they are a minority crop. *L. albus* readily forms CR under moderate P deficiency, Fe deficiency or Al toxicity, while some other cultivated lupins are capable of producing CR under harsher stress conditions (Pueyo et al., 2021), which implies that they probably share Al-detoxification systems. While *L. albus* is the most studied species in terms of tolerance mechanisms, other *Lupinus* species are also quite tolerant to acidic soils and Al toxicity. CR formation is triggered by specific soil conditions, but the mechanisms involved are similar to those known to control growth of regular roots. CR provide an expanded surface to interact with the rhizospheric soil. Lupins establish symbiosis with soil bacteria, mostly within the *Bradyrhizobium* genus, which contribute to Al immobilization. Al tolerance mechanisms in lupin are common to other Al-tolerant plants, but specific features derived from the root structural modifications and the interaction with symbiotic bacteria are outstanding. Here, we have analyzed the existing knowledge on the particular mechanisms that control lupin tolerance to Al. Citrate exudation induced by Al in lupin is not coupled with H^+^ secretion through plasma membrane H^+^-ATPase, and when Al toxicity is concomitant with P deficiency, a K^+^ efflux occurs that is not caused by Al stress alone (Zeng et al., 2013). Contrary to some homologs in other plants, LaALMT1 expression is not stimulated by Al, and it is not involved in malate exudation. The transport mechanisms in Al-induced citrate secretion are not completely understood and need further investigation. This research line has not really progressed in the last few years; however, the fully white lupin sequenced genome and some novel technologies, including omics and advanced transformation techniques, provide new tools to elucidate the specific Al-triggered mechanisms that lead to citrate (and other less studied metabolites) exudation.

While different rhizobacteria can help reduce Al stress, the slow-growing bradyrhizobia that nodulate lupin have been reported to produce abundant exopolysaccharides, which immobilize metal ions. Lupin tolerance mechanisms have been investigated and deciphered to some extent; however, little is known on the accumulation of metals by lupin. Rooibos tea (*Aspalathus linearis*) is a legume that also grows in acidic, Al-rich, nutrient-poor soils (Kanu et al., 2013). Like lupin, it forms CR that exude Al-chelating molecules, and it accumulates Al–Si complexes (Kanu et al., 2013). It has been proposed as a suitable candidate for phytoremediation. Recently, white lupin has been reported as an Hg-resistant plant capable of Hg rhizosequestration, especially in CR, with null translocation of the metal to the aerial parts of the plant (Quiñones et al., 2021). It appears that CR have a role not only in elevated secretion of metal chelators, but also in the immobilization of toxic metals, a subject that certainly requires further investigation for its practical application in avoiding translocation to the aerial parts of the plant, a topic that requires further analyses, and for the elucidation of metal sequestration mechanisms in plant roots.

White lupin in symbiosis with tolerant bradyrhizobia represents a potentially powerful tool for metal-polluted soil phytoremediation and degraded landscape restoration, entailing significant environmental benefits. Moreover, lupin cultivation involves an enrichment of the soils with N through atmospheric N_2_ fixation, and nutrients such as P and Fe through CR exudates-mediated solubilization that may be taken advantage of by rotating crops. In conclusion, lupin cultivation as a protein crop arises as an optimal environment-friendly alternative to exploit acidic soils in temperate zones affected by Al toxicity, where other crops might not be sufficiently productive to be economically viable.

## Data Availability Statement

The original contributions presented in the study are included in the article/supplementary material, further inquiries can be directed to the corresponding author/s.

## Author Contributions

JJP conceived the work and designed the figures. JJP and MAQ wrote the first draft. MAQ, MML, and JJP contributed to the writing of the manuscript, proofread, and approved its content.

## Conflict of Interest

The authors declare that the research was conducted in the absence of any commercial or financial relationships that could be construed as a potential conflict of interest.

## Publisher’s Note

All claims expressed in this article are solely those of the authors and do not necessarily represent those of their affiliated organizations, or those of the publisher, the editors and the reviewers. Any product that may be evaluated in this article, or claim that may be made by its manufacturer, is not guaranteed or endorsed by the publisher.

## References

[B1] Andersson M. (1988). Toxicity and tolerance of aluminium in vascular plants – a literature review. *Water Air Soil Pollut.* 39 439–462. 10.1007/BF00279487

[B2] Arora N. K. Khare E. Singh S. Maheshwari D. K. (2010). Effect of Al and heavy metals on enzymes of nitrogen metabolism of fast and slow growing rhizobia under explanta conditions. *World J. Microbiol. Biotechnol.* 26 811–816. 10.1007/s11274-009-0237-6

[B3] Arregui G. Hipólito P. Pallol B. Lara-Dampier V. García-Rodríguez D. Varela H. P. (2021). Mercury-tolerant Ensifer medicae strains display high mercuric reductase activity and a protective effect on nitrogen fixation in *Medicago truncatula* nodules under mercury stress. *Front. Plant Sci.* 11:560768. 10.3389/fpls.2020.560768 33519831 PMC7840509

[B4] Beck M. A. Robarge W. P. Buol S. W. (1999). Phosphorus retention and release of anions and organic carbon by two Andisols. *Eur. J. Soil Sci.* 50 157–164. 10.1046/j.1365-2389.1999.00213.x

[B5] Billou I. Xu J. Wildwater M. Willemsen V. Paponov I. Frimi J. (2005). The PIN auxin efflux facilitator network controls growth and patterning in *Arabidopsis* roots. *Nature* 433 39–44. 10.1038/nature03184 15635403

[B6] Burzyński A. Piślewska M. Wojtaszek P. (2000). Active chitinases in the apoplastic fluids of healthy white lupin (*Lupinus albus* L.) plants. *Acta Physiol. Plant.* 22 31–38. 10.1007/s11738-000-0005-6

[B7] Carlsbecker A. Lee J. Y. Roberts C. J. Dettmer J. Lehesranta S. Zhou J. (2010). Cell signalling by microRNA165/6 directs gene dose-dependent root cell fate. *Nature* 465 316–321. 10.1038/nature08977 20410882 PMC2967782

[B8] Carr J. Ritchie P. (1993). Al toxicity of wheat grown in acidic subsoils in relation to soil solution properties and exchangeable cations. *Aust. J. Soil Res.* 31 583–596. 10.1071/sr9930583

[B9] Cesco S. Neumann G. Tomasi N. Pinton R. Weisskopf L. (2010). Release of plant-borne flavonoids into the rhizosphere and their role in plant nutrition. *Plant Soil* 329 1–25. 10.1007/s11104-009-0266-9

[B10] Chauhan D. K. Yadav V. Vaculík M. Gassmann W. Pike S. Arif N. (2021). Aluminum toxicity and aluminum stress-induced physiological tolerance responses in higher plants. *Crit. Rev. Biotechnol.* 41 715–730. 10.1080/07388551.2021.1874282 33866893

[B11] Chen H. Richardson A. E. Rolfe B. G. (1993). Studies of the physiological and genetic basis of acid tolerance in *Rhizobium leguminosarum* biovar *trifolii*. *Appl. Environ. Microbiol.* 59 1798–1804. 10.1128/aem.59.6.1798-1804.1993 16348956 PMC182164

[B12] Chen Z. C. Liao H. (2016). Organic acid anions: an effective defensive weapon for plants against aluminum toxicity and phosphorus deficiency in acidic soils. *J. Genet. Genomics* 43 631–638. 10.1016/j.jgg.2016.11.003 27890545

[B13] Coba de la Peña T. Pueyo J. J. (2012). Legumes in the reclamation of marginal soils, from cultivar and inoculant selection to transgenic approaches. *Agron. Sustain. Dev.* 32 65–91. 10.1007/s13593-011-0024-2

[B14] Coba de la Peña T. Fedorova E. Pueyo J. J. Lucas M. M. (2018). The symbiosome: legume and rhizobia co-evolution toward a nitrogen-fixing organelle? *Front. Plant Sci.* 8:2229. 10.3389/fpls.2017.02229 29403508 PMC5786577

[B15] Cooper J. E. (2007). Early interactions between legumes and rhizobia: disclosing complexity in a molecular dialogue. *J. Appl. Microbiol.* 103 1355–1365. 10.1111/j.1365-2672.2007.03366.x 17953546

[B16] Coudert Y. Périn C. Courtois B. Khong N. G. Gantet P. (2010). Genetic control of root development in rice, the model cereal. *Trends Plant Sci.* 15 219–226. 10.1016/j.tplants.2010.01.008 20153971

[B17] Dakora F. D. Phillips D. A. (2002). Root exudates as mediators of mineral acquisition in low-nutrient environments. *Plant Soil* 245 35–47. 10.1023/A:1020809400075

[B18] De Ron A. M. Sparvoli F. Pueyo J. J. Bazile D. (2017). Protein crops: food and feed for the future. *Front. Plant Sci.* 8:105. 10.3389/fpls.2017.00105 28220133 PMC5292564

[B19] Dessureault-Rompré J. Nowack B. Schulin R. Luster J. (2007). Spatial and temporal variation in organic acid anion exudation and nutrient anion uptake in the rhizosphere of *Lupinus albus* L. *Plant Soil* 301 123–134. 10.1007/s11104-007-9427-x

[B20] Dessureault-Rompré J. Nowack B. Schulin R. Tercier-Waeber M. L. Luster J. (2008). Metal solubility and speciation in the rhizosphere of *Lupinus albus* cluster roots. *Environ. Sci. Technol.* 42 7146–7151. 10.1021/es800167g 18939539

[B21] Dong D. Peng X. Yan X. (2004). Organic acid exudation induced by phosphorus deficiency and/or aluminium toxicity in two contrasting soybean genotypes. *Physiol. Plant.* 122 190–199. 10.1111/j.1399-3054.2004.00373.x

[B22] Eriksson A. K. Hillier S. Hesterberg D. Klysubun W. Ulén B. Gustafsson J. P. (2016). Evolution of phosphorus speciation with depth in an agricultural soil profile. *Geoderma* 280 29–37. 10.1016/j.geoderma.2016.06.004

[B23] Fedorova E. E. De Felipe M. R. Pueyo J. J. Lucas M. M. (2007). Conformation of cytoskeletal elements during the division of infected *Lupinus albus* L. nodule cells. *J. Exp. Bot.* 58 2225–2236. 10.1093/jxb/erm083 17525079

[B24] Fernández-Pascual M. Pueyo J. J. Felipe M. R. Golvano M. P. Lucas M. M. (2007). Singular features of the *Bradyrhizobium*-*Lupinus* symbiosis. *Dyn. Soil Dyn. Plant* 1 1–16. 10.1007/978-3-319-23534-9_1

[B25] Ferreira P. A. A. Bomfeti C. A. Soares B. L. de Souza Moreira F. M. (2012). Efficient nitrogen-fixing *Rhizobium* strains isolated from amazonian soils are highly tolerant to acidity and aluminium. *World J. Microbiol. Biotechnol.* 28 1947–1959. 10.1007/s11274-011-0997-7 22806016

[B26] Flis S. E. Glenn A. R. Dilworth M. J. (1993). The interaction between aluminium and root nodule bacteria. *Soil Biol. Biochem.* 25 403–417. 10.1016/0038-0717(93)90066-K

[B27] Galinha C. Hofhuis H. Luijten M. Willemsen V. Blilou I. Heidstra R. (2007). PLETHORA proteins as dose-dependent master regulators of *Arabidopsis* root development. *Nature* 449 1053–1057. 10.1038/nature06206 17960244

[B28] Geelhoed J. S. Hiemstra T. Van Riemsdijk W. H. (1998). Competitive interaction between phosphate and citrate on goethite. *Environ. Sci. Technol.* 32 2119–2123. 10.1021/es970908y

[B29] Gerke J. Römer W. Jungk A. (1994). The excretion of citric and malic acid by proteoid roots of *Lupinus albus* L.; effects on soil solution concentrations of phosphate, iron, and aluminum in the proteoid rhizosphere in samples of an oxisol and a luvisol. *Z. Pflanzenernährung Bodenkd.* 157 289–294. 10.1002/jpln.19941570408

[B30] Gilbert G. A. Knight J. D. Vance C. P. Allan D. L. (1999). Acid phosphatase activity in phosphorus-deficient white lupin roots. *Plant Cell Environ.* 22 801–810. 10.1046/j.1365-3040.1999.00441.x

[B31] González-Sama A. De La Peña T. C. Kevei Z. Mergaert P. Lucas M. M. De Felipe M. R. (2006). Nuclear DNA endoreduplication and expression of the mitotic inhibitor Ccs52 associated to determinate and lupinoid nodule organogenesis. *Mol. Plant Microbe Interact.* 19 173–180. 10.1094/MPMI-19-0173 16529379

[B32] González-Sama A. Lucas M. M. De Felipe M. R. Pueyo J. J. (2004). An unusual infection mechanism and nodule morphogenesis in white lupin (*Lupinus albus*). *New Phytol.* 163 371–380. 10.1111/j.1469-8137.2004.01121.x 33873628

[B33] Grieneisen V. A. Xu J. Marée A. F. M. Hogeweg P. Scheres B. (2007). Auxin transport is sufficient to generate a maximum and gradient guiding root growth. *Nature* 449 1008–1013. 10.1038/nature06215 17960234

[B34] Guo C. L. Chen Q. Zhao X. L. Chen X. Q. Zhao Y. Wang L. (2013). Al-enhanced expression and interaction of 14-3-3 protein and plasma membrane H^+^-ATPase is related to Al-induced citrate secretion in an Al-resistant black soybean. *Plant Mol. Biol. Report.* 31 1012–1024. 10.1007/s11105-013-0569-0

[B35] Hemada M. Mohamed H. E. Saber N. E.-S. El-Ekeili A. M. (2020). Role of salicylic acid in alleviation of aluminum effects on growth and biochemical processes in lupin (*Lupinus termis* L.) plant. *Egypt. J. Bot.* 60 287–300. 10.21608/ejbo.2019.15177.1343

[B36] Igamberdiev A. U. Eprintsev A. T. (2016). Organic acids: the pools of fixed carbon involved in redox regulation and energy balance in higher plants. *Front. Plant Sci.* 7:1042. 10.3389/fpls.2016.01042 27471516 PMC4945632

[B37] Jaiswal S. K. Naamala J. Dakora F. D. (2018). Nature and mechanisms of aluminium toxicity, tolerance and amelioration in symbiotic legumes and rhizobia. *Biol. Fertil. Soils* 54 309–318. 10.1007/s00374-018-1262-0 31258230 PMC6560468

[B38] Jansen P. C. M. (2006). “*Lupinus albus* L,” in *Record from PROTA4U*, eds Brink M. Belay G. (Wageningen: PROTA (Plant Resources of Tropical Africa/Ressources végétales de l’Afrique Tropicale)).

[B39] Johnson J. F. Allan D. L. Vance C. P. (1994). Phosphorus stress-induced proteoid roots show altered metabolism in *Lupinus albus.* *Plant Physiol.* 104, 657–665. 10.1104/pp.104.2.657 12232116 PMC159244

[B40] Kaiser K. Zech W. (1997). Competitive sorption of dissolved organic matter fractions to soils and related mineral phases. *Soil Sci. Soc. Am. J.* 61 64–69. 10.2136/sssaj1997.03615995006100010011x

[B41] Kang J. P. Huo Y. Yang D. U. Yang D. C. (2021). Influence of the plant growth promoting *Rhizobium panacihumi* on aluminum resistance in *Panax ginseng*. *J. Ginseng Res.* 45 442–449. 10.1016/j.jgr.2020.01.001 34025137 PMC8134844

[B42] Kanu S. A. Okonkwo J. O. Dakora F. D. (2013). *Aspalathus linearis* (Rooibos tea) as potential phytoremediation agent: a review on tolerance mechanisms for aluminum uptake. *Environ. Rev.* 21 85–92. 10.1139/er-2012-0055

[B43] Lambers H. Cramer M. D. Shane M. W. Wouterlood M. Poot P. Veneklaas E. J. (2003). Structure and functioning of cluster roots and plant responses to phosphate deficiency. *Plant Soil* 248 9–19. 10.1023/A:1025561812696

[B44] Lamont B. (2003). Structure, ecology and physiology of root clusters – a review. *Plant Soil* 248 1–19. 10.1023/A:1022314613217

[B45] Lesueur D. Diem H. G. Dianda M. Le Roux C. (1993). Selection of *Bradyrhizobium* strains and provenances of *Acacia mangium* and *Faidherbia albida*: relationship with their tolerance to acidity and aluminium. *Plant Soil* 149 159–166. 10.1007/BF00016605

[B46] Ligaba A. Yamaguchi M. Shen H. Sasaki T. Yamamoto Y. Matsumoto H. (2004). Phosphorus deficiency enhances plasma membrane H^+^-ATPase activity and citrate exudation in greater purple lupin (*Lupinus pilosus*). *Funct. Plant Biol.* 31 1075–1083. 10.1071/FP04091 32688975

[B47] Liu P. P. Montgomery T. A. Fahlgren N. Kasschau K. D. Nonogaki H. Carrington J. C. (2007). Repression of AUXIN response factor10 by microRNA160 is critical for seed germination and post-germination stages. *Plant J.* 52 133–146. 10.1111/j.1365-313X.2007.03218.x 17672844

[B48] Lucas M. M. Stoddard F. L. Annicchiarico P. Frías J. Martínez-Villaluenga C. Sussmann D. (2015). The future of lupin as a protein crop in Europe. *Front. Plant Sci.* 6:705. 10.3389/fpls.2015.00705 26442020 PMC4561814

[B49] Lv A. Wen W. Fan N. Su L. Zhou P. An Y. (2021). Dehydrin MsDHN1 improves aluminum tolerance of alfalfa (*Medicago sativa* L.) by affecting oxalate exudation from root tips. *Plant J.* 108 441–458. 10.1111/tpj.15451 34363255

[B50] Ma J. F. (2000). Role of organic acids in detoxification of aluminum in higher plants. *Plant Cell Physiol.* 41 383–390. 10.1093/pcp/41.4.383 10845450

[B51] Ma J. F. Ryan P. R. Delhaize E. (2001). Aluminium tolerance in plants and the complexing role of organic acids. *Trends Plant Sci.* 6 273–278. 10.1016/S1360-1385(01)01961-6 11378470

[B52] Matsumoto H. (2000). Cell biology of aluminum toxicity tolerance in higher plants. *Int. Rev. Cytol.* 200 1–46. 10.1016/s0074-7696(00)00001-2 10965465

[B53] Meng Z. B. You X. D. Suo D. Chen Y. L. Tang C. Yang J. L. (2013). Root-derived auxin contributes to the phosphorus-deficiency-induced cluster-root formation in white lupin (*Lupinus albus*). *Physiol. Plant.* 148 481–489. 10.1111/j.1399-3054.2012.01715.x 23067249

[B54] Mimmo T. Ghizzi M. Marzadori C. Gessa C. E. (2008). Organic acid extraction from rhizosphere soil: effect of field-moist, dried and frozen samples. *Plant Soil* 312 175–184. 10.1007/s11104-008-9574-8

[B55] Moore G. Dolling P. Porter B. Leonard L. (2001). “Chemical factors affecting plant growth. Soil acidity,” in *Soil Guide. A Handbook for Undersanding and Managing Agricultural Soils*, eds Moore G. McFarlane J. Purdie B. Wilson G. (Perth, WA: Department of Agriculture and Food), 127–140.

[B56] Msaddak A. Rey L. Imperial J. Palacios J. M. Mars M. Pueyo J. J. (2021). Phylogenetic analyses of rhizobia isolated from nodules of *Lupinus angustifolius* in Northern Tunisia reveal *Devosia* sp. As a new microsymbiont of lupin species. *Agronomy* 11:1510. 10.3390/agronomy11081510

[B57] Nelson P. Moore G. Dracup M. (2001). “Crops: soil and climatc requirements. Narrow-leafed lupins,” in *Soil Guide. A Handbook for Undersanding and Managing Agricultural Soils*, eds Moore G. McFarlane J. Purdie B. Wilson G. (Perth, WA: Department of Agriculture and Food), 272–273. 10.1111/nph.14094

[B58] Neumann G. Massonneau A. Martinoia E. Römheld V. (1999). Physiological adaptations to phosphorus deficiency during proteoid root development in white lupin. *Planta* 208 373–382. 10.1007/s004250050572

[B59] Panagos P. Van Liedekerke M. Jones A. Montanarella L. (2012). European soil data centre: response to European policy support and public data requirements. *Land Use Policy* 29 329–338. 10.1016/j.landusepol.2011.07.003

[B60] Petricka J. J. Winter C. M. Benfey P. N. (2012). Control of *Arabidopsis* root development. *Annu. Rev. Plant Biol.* 63 563–590. 10.1146/annurev-arplant-042811-105501 22404466 PMC3646660

[B61] Pueyo J. J. Quiñones M. A. Coba de la Peña T. Fedorova E. E. Lucas M. M. (2021). Nitrogen and phosphorus interplay in lupin root nodules and cluster roots. *Front. Plant Sci.* 12:644218. 10.3389/fpls.2021.644218 33747024 PMC7966414

[B62] Pulsawat W. Leksawasdi N. Rogers P. L. Foster L. J. R. (2003). Anions effects on biosorption of Mn(II) by extracellular polymeric substance (EPS) from *Rhizobium etli*. *Biotechnol. Lett.* 25 1267–1270. 10.1023/A:1025083116343 14514079

[B63] Quiñones M. A. Fajardo S. Fernández-Pascual M. Lucas M. M. Pueyo J. J. (2021). Nodulated white lupin plants growing in contaminated soils accumulate unusually high mercury concentrations in their nodules, roots and especially cluster roots. *Horticulturae* 7 1–19. 10.3390/horticulturae7090302

[B64] Quiñones M. A. Ruiz-Díez B. Fajardo S. López-Berdonces M. A. Higueras P. L. Fernández-Pascual M. (2013). *Lupinus albus* plants acquire mercury tolerance when inoculated with an Hg-resistant *Bradyrhizobium* strain. *Plant Physiol. Biochem.* 73 168–175. 10.1016/j.plaphy.2013.09.015 24125840

[B65] Rademacher E. H. Möller B. Lokerse A. S. Llavata-Peris C. I. Van Den Berg W. Weijers D. (2011). A cellular expression map of the *Arabidopsis* AUXIN response factor gene family. *Plant J.* 68 597–606. 10.1111/j.1365-313X.2011.04710.x 21831209

[B66] Rajkumar M. Ae N. Prasad M. N. V. Freitas H. (2010). Potential of siderophore-producing bacteria for improving heavy metal phytoextraction. *Trends Biotechnol.* 28 142–149. 10.1016/j.tibtech.2009.12.002 20044160

[B67] Rangel A. F. Rao I. M. Horst W. J. (2007). Spatial aluminium sensitivity of root apices of two common bean (*Phaseolus vulgaris* L.) genotypes with contrasting aluminium resistance. *J. Exp. Bot.* 58 3895–3904. 10.1093/jxb/erm241 17975208

[B68] Redel Y. Cartes P. Velásquez G. Poblete-Grant P. Poblete-Grant P. Bol R. (2016). Assessment of phosphorus status influenced by Al and Fe compounds in volcanic grassland soils. *J. Soil Sci. Plant Nutr.* 16 490–506. 10.4067/S0718-95162016005000041 27315006

[B69] Roy N. Chakrabartty P. K. (2000). Effect of aluminum on the production of siderophore by *Rhizobium* sp. (*Cicer arietinum*). *Curr. Microbiol.* 41 5–10. 10.1007/s002840010082 10919391

[B70] Rudrappa T. Czymmek K. J. Paré P. W. Bais H. P. (2008). Root-secreted malic acid recruits beneficial soil bacteria. *Plant Physiol.* 148 1547–1556. 10.1104/pp.108.127613 18820082 PMC2577262

[B71] Sade H. Meriga B. Surapu V. Gadi J. Sunita M. S. L. Suravajhala P. (2016). Toxicity and tolerance of aluminum in plants: tailoring plants to suit to acid soils. *BioMetals* 29 187–210. 10.1007/s10534-016-9910-z 26796895

[B72] Secco D. Shou H. Whelan J. Berkowitz O. (2014). RNA-seq analysis identifies an intricate regulatory network controlling cluster root development in white lupin. *BMC Genomics* 15:230. 10.1186/1471-2164-15-230 24666749 PMC4028058

[B73] Sharma T. Dreyer I. Kochian L. Piñeros M. A. (2016). The ALMT family of organic acid transporters in plants and their involvement in detoxification and nutrient security. *Front. Plant Sci.* 7:1488. 10.3389/fpls.2016.01488 27757118 PMC5047901

[B74] Shen H. He L. F. Sasaki T. Yamamoto Y. Zheng S. J. Ligaba A. (2005). Citrate secretion coupled with the modulation of soybean root tip under aluminum stress. Up-regulation of transcription, translation, and threonine-oriented phosphorylation of plasma membrane H^+^-ATPase. *Plant Physiol.* 138 287–296. 10.1104/pp.104.900170 15834009 PMC1104183

[B75] Sprent J. I. (2009). *Legume Nodulation. A Global Perspective.* Chichester: Wiley-Blackwell, 10.1016/j.cub.2014.01.028

[B76] Sun L. Tian J. Zhang H. Liao H. (2016). Phytohormone regulation of root growth triggered by P deficiency or Al toxicity. *J. Exp. Bot.* 67 3655–3664. 10.1093/jxb/erw188 27190050

[B77] Sun P. Tian Q. Y. Zhao M. G. Dai X. Y. Huang J. H. Li L. H. (2007). Aluminum-induced ethylene production is associated with inhibition of root elongation in *Lotus japonicus* L. *Plant Cell Physiol.* 48 1229–1235. 10.1093/pcp/pcm077 17573361

[B78] Swarup K. Benková E. Swarup R. Casimiro I. Péret B. Yang Y. (2008). The auxin influx carrier LAX3 promotes lateral root emergence. *Nat. Cell Biol.* 10 946–954. 10.1038/ncb1754 18622388

[B79] Swarup R. Perry P. Hagenbeek D. Van Der Straeten D. Beemster G. T. S. Sandberg G. (2007). Ethylene upregulates auxin biosynthesis in *Arabidopsis* seedlings to enhance inhibition of root cell elongation. *Plant Cell* 19 2186–2196. 10.1105/tpc.107.052100 17630275 PMC1955695

[B80] Tomasi N. Kretzschmar T. Espen L. Weisskopf L. Fuglsang A. T. Palmgren M. G. (2009). Plasma membrane H^+^-ATPase-dependent citrate exudation from cluster roots of phosphate-deficient white lupin. *Plant Cell Environ.* 32 465–475. 10.1111/j.1365-3040.2009.01938.x 19183296

[B81] Uhde-Stone C. Gilbert G. Johnson J. M. F. Litjens R. Zinn K. E. Temple S. J. (2003). Acclimation of white lupin to phosphorus deficiency involves enhanced expression of genes related to organic acid metabolism. *Plant Soil* 248 99–116. 10.1023/A:1022335519879

[B82] Vanneste S. Friml J. (2009). Auxin: a trigger for change in plant development. *Cell* 136 1005–1016. 10.1016/j.cell.2009.03.001 19303845

[B83] Vargas L. K. Lisboa B. B. Scholles D. Silveira J. R. P. Jung G. C. Granada C. E. (2007). Genetic diversity and symbiotic efficiency of black wattle-nodulating rhizobia in soils of Rio Grande do Sul State, Brazil. *Rev. Bras. Cienc. Solo* 31 647–654. 10.1590/s0100-06832007000400005

[B84] Violante A. Colombo C. Buondonno A. (1991). Competitive adsorption of phosphate and oxalate by aluminum oxides. *Soil Sci. Soc. Am. J.* 55 65–70. 10.2136/sssaj1991.03615995005500010011x

[B85] Wang B. L. Shen J. B. Zhang W. H. Zhang F. S. Neumann G. (2007). Citrate exudation from white lupin induced by phosphorus deficiency differs from that induced by aluminum. *New Phytol.* 176 581–589. 10.1111/j.1469-8137.2007.02206.x 17725555

[B86] Wang J. W. Wang L. J. Mao Y. B. Cai W. J. Xue H. W. Chen X. Y. (2005). Control of root cap formation by MicroRNA-targeted auxin response factors in *Arabidopsis*. *Plant Cell* 17 2204–2216. 10.1105/tpc.105.033076 16006581 PMC1182483

[B87] Wang J. Hou Q. Li P. Yang L. Sun X. Benedito V. A. (2017). Diverse functions of multidrug and toxin extrusion (MATE) transporters in citric acid efflux and metal homeostasis in *Medicago truncatula*. *Plant J.* 90 79–95. 10.1111/tpj.13471 28052433

[B88] Wei Y. Han R. Xie Y. Jiang C. Yu Y. (2021). Recent advances in understanding mechanisms of plant tolerance and response to aluminum toxicity. *Sustainability* 13:1782. 10.3390/su13041782

[B89] Weisskopf L. Tomasi N. Santelia D. Martinoia E. Langlade N. B. Tabacchi R. (2006). Isoflavonoid exudation from white lupin roots is influenced by phosphate supply, root type and cluster-root stage. *New Phytol.* 171 657–668. 10.1111/j.1469-8137.2006.01776.x 16866966

[B90] Xu M. You J. Hou N. Zhang H. Chen G. Yang Z. (2010). Mitochondrial enzymes and citrate transporter contribute to the aluminium-induced citrate secretion from soybean (*Glycine max*) roots. *Funct. Plant Biol.* 37 285–295. 10.1071/FP09223

[B91] Yang J. L. Zhu X. F. Peng Y. X. Zheng C. Ming F. Zheng S. J. (2011). Aluminum regulates oxalate secretion and plasma membrane H^+^-ATPase activity independently in tomato roots. *Planta* 234 281–291. 10.1007/s00425-011-1402-3 21424534

[B92] Yang Z. B. Geng X. He C. Zhang F. Wang R. Horst W. J. (2014). TAA1-regulated local auxin biosynthesis in the root-apex transition zone mediates the aluminum-induced inhibition of root growth in *Arabidopsis*. *Plant Cell* 26 2889–2904. 10.1105/tpc.114.127993 25052716 PMC4145121

[B93] Zavalin A. A. Sokolov O. A. Shmyreva N. Y. Lukin S. V. (2019). Legume reaction to soil acidity. *Amaz. Investig.* 8 162–170.

[B94] Zeng H. Feng X. Wang B. Zhu Y. Shen Q. Xu G. (2013). Citrate exudation induced by aluminum is independent of plasma membrane H^+^-ATPase activity and coupled with potassium efflux from cluster roots of phosphorus-deficient white lupin. *Plant Soil* 366 389–400. 10.1007/s11104-012-1445-7

[B95] Zheng S. J. (2010). Crop production on acidic soils: overcoming aluminium toxicity and phosphorus deficiency. *Ann. Bot.* 106 183–184. 10.1093/aob/mcq134 20570831 PMC2889811

[B96] Zhou Y. Neuhäuser B. Neumann G. Ludewig U. (2020). LaALMT1 mediates malate release from phosphorus-deficient white lupin root tips and metal root to shoot translocation. *Plant Cell Environ.* 43 1691–1706. 10.1111/pce.13762 32239684

[B97] Zhou Y. Yang Z. Xu Y. Sun H. Sun Z. Lin B. (2018). Soybean NADP-malic enzyme functions in malate and citrate metabolism and contributes to their efflux under Al stress. *Front. Plant Sci.* 8:2246. 10.3389/fpls.2017.02246 29367856 PMC5767732

[B98] Zhu Y. Yan F. Zörb C. Schubert S. (2005). A link between citrate and proton release by proteoid roots of white lupin (*Lupinus albus* L.) grown under phosphorus-deficient conditions? *Plant Cell Physiol.* 46 892–901. 10.1093/pcp/pci094 15821025

